# Novel Anti-MRSA Peptide from Mangrove-Derived *Virgibacillus chiguensis* FN33 Supported by Genomics and Molecular Dynamics

**DOI:** 10.3390/md23050209

**Published:** 2025-05-14

**Authors:** Namfa Sermkaew, Apichart Atipairin, Phetcharat Boonruamkaew, Sucheewin Krobthong, Chanat Aonbangkhen, Jumpei Uchiyama, Yodying Yingchutrakul, Nuttapon Songnaka

**Affiliations:** 1 School of Pharmacy, Walailak University, Thasala, Nakhon Si Thammarat 80160, Thailand; namfa.se@wu.ac.th (N.S.); apichart.at@mail.wu.ac.th (A.A.); phetcharat.bo@wu.ac.th (P.B.); 2 Drug and Cosmetics Excellence Center, Walailak University, Thasala, Nakhon Si Thammarat 80160, Thailand; 3 Center of Excellence in Natural Products Chemistry (CENP), Department of Chemistry, Faculty of Science, Chulalongkorn University, Bangkok 10330, Thailand; sucheewin.k@chula.ac.th (S.K.); chanat.a@chula.ac.th (C.A.); 4 Center of Excellence on Petrochemical and Materials Technology, Chulalongkorn University, Bangkok 10330, Thailand; 5 Department of Bacteriology, Graduate School of Medicine, Dentistry and Pharmaceutical Sciences, Okayama University, Okayama 700-8558, Japan; uchiyama@okayama-u.ac.jp; 6 National Center for Genetic Engineering and Biotechnology, National Science and Technology Development Agency, Pathum Thani 12120, Thailand; yodying.yin@biotec.or.th

**Keywords:** anionic AMP, AMP, antimicrobial peptide, antimicrobial resistance, FN33, genome, molecular dynamics simulations, MRSA, *Virgibacillus chiguensis*

## Abstract

Antimicrobial resistance (AMR) is a global health threat, with methicillin-resistant *Staphylococcus aureus* (MRSA) being one of the major resistant pathogens. This study reports the isolation of a novel mangrove-derived bacterium, *Virgibacillus chiguensis* FN33, as identified through genome analysis and the discovery of a new anionic antimicrobial peptide (AMP) exhibiting anti-MRSA activity. The AMP was composed of 23 amino acids, which were elucidated as NH_3_-Glu-Gly-Gly-Cys-Gly-Val-Asp-Thr-Trp-Gly-Cys-Leu-Thr-Pro-Cys-His-Cys-Asp-Leu-Phe-Cys-Thr-Thr-COOH. The minimum inhibitory concentration (MIC) and minimum bactericidal concentration (MBC) for MRSA were 8 µg/mL and 16 µg/mL, respectively. FN33 AMP induced cell membrane permeabilization, suggesting a membrane-disrupting mechanism. The AMP remained stable at 30–40 °C but lost activity at higher temperatures and following exposure to proteases, surfactants, and extreme pH. All-atom molecular dynamics simulations showed that the AMP adopts a β-sheet structure upon membrane interaction. These findings suggest that *Virgibacillus chiguensis* FN33 is a promising source of novel antibacterial agents against MRSA, supporting alternative strategies for drug-resistant infections.

## 1. Introduction

Antimicrobial resistance (AMR) occurs when microorganisms evolve to resist antimicrobial agents such as antibiotics. This issue has become one of the most pressing global health concerns of the 21st century due to the rapid rise in AMR infections and the limited development of new antimicrobial agents. A significant contributing factor to AMR is the overuse and misuse of antibiotics across clinical settings, agriculture, animal healthcare, and the food industry [1].

In 2019, bacterial AMR was responsible for 1.27 million deaths, with methicillin-resistant *Staphylococcus aureus* (MRSA) being a major cause of this mortality [2]. MRSA is associated with a range of severe infections, including pneumonia, pleuritis, tympanitis, purulent meningitis, and bloodstream infections, leading to prolonged hospital stays and increased mortality rates in patients with bacteremia [3,4]. The high and rapidly increasing prevalence of MRSA worldwide underscores the urgent need for new antimicrobial agents that can effectively combat resistant bacterial strains [5].

The mangrove ecosystem represents a promising yet underexplored source of bioactive compounds due to its unique environmental conditions, such as high tides, hypersaline waters, and significant temperature fluctuations. These extreme conditions create a challenging habitat that fosters the survival of adaptable microorganisms capable of producing antimicrobial compounds [6,7].

One such microbial genus, *Virgibacillus*, belongs to the Bacillaceae family in the Firmicutes phylum and is predominantly found in saline environments [8]. Members of this genus are rod-shaped, endospore-forming, and Gram-stain variable or Gram-positive. Due to their ability to thrive in diverse environments, including marine sediments [9], several species of *Virgibacillus* have been reported to exhibit antimicrobial activity, particularly against *S. aureus* [10,11,12]. Notably, *Virgibacillus salarius* has demonstrated antibacterial activity against MRSA [11,13]. Given the potential of mangrove sediments as a reservoir for antimicrobial-producing bacteria [14], this study focused on isolating and characterizing a new *Virgibacillus* strain from mangrove sediments as a potential producer of novel antimicrobial peptides (AMPs).

AMPs represent a promising class of alternative antimicrobial agents with the potential to combat resistant bacterial infections. Unlike conventional antibiotics, AMPs disrupt bacterial membranes through direct interactions, increasing permeability and forming pores, ultimately leading to cell death [15]. A major group of AMPs consists of linear peptides ranging from 12 to 50 amino acids, which have naturally evolved as part of bacterial defense mechanisms and the innate immune system of multicellular organisms [16].

AMPs are predominantly composed of cationic and hydrophobic amino acids, allowing them to selectively interact with negatively charged bacterial membranes via electrostatic interactions [17]. Traditionally, AMPs have been classified based on their net charge, with cationic AMPs being rich in arginine or lysine and anionic AMPs containing aspartic acid or glutamic acid. Cationic AMPs have been widely studied due to their strong electrostatic attraction to the negatively charged microbial cell surface, a key factor in their antimicrobial activity. This charge-dependent mechanism initially led to the assumption that anionic AMPs would be less effective due to the lack of strong charge-based attraction. However, research has shown that anionic AMPs, like their cationic counterparts, can adopt amphiphilic α-helices and β-sheets, allowing them to interact with microbial membranes [18]. Several anionic AMPs have been identified, such as maximin H5 from amphibians, which has demonstrated activity against *S. aureus* [17], and dermcidin, a human-derived anionic AMP [19]. Therefore, anionic AMPs are gaining attention as potential therapeutic alternatives, offering new possibilities for combating AMR and making them a promising area of study [20].

Molecular dynamics (MD) simulations have been widely utilized in protein research, ranging from analyzing their basic motions in solution to simulating the folding process. More recently, MD has played a crucial role in drug discovery, driven by advancements in computational power [21]. Determining the three-dimensional structures of peptides is essential for understanding their function and secondary structure. Numerous MD simulations are conducted to investigate peptide–lipid bilayer interactions, providing insights into structural dynamics and membrane disruption mechanisms [21,22].

This study aimed to isolate and characterize a novel anionic AMP from *Virgibacillus chiguensis* FN33 with activity against MRSA, guided by genome-based analysis of bio-synthetic gene clusters. It also explored the peptide’s interaction with bacterial membranes through molecular dynamics simulations. The findings support the potential of FN33 AMP as an alternative treatment for drug-resistant bacterial infections.

## 2. Results

### 2.1. Investigating the Antimicrobial Potential of Bacteria in Mangrove Sediments

Sediment samples were collected from the mangrove region, Nakhon Si Thammarat, Thailand. The bacterial colonies isolated from Mueller–Hinton (MH) agar, Zobell Marine (ZM) agar, and Starch Casein (SC) agar were screened for the activity using the soft agar overlay method against MRSA strain 2468. The soft agar overlay assay revealed that only one isolate, which was designated FN33, exhibited antimicrobial activity against MRSA strain 2468. This culture was further subcultured in liquid medium to obtain a cell-free supernatant (CFS) for subsequent analysis. The antibacterial spectrum was assessed using the agar well diffusion assay against various pathogens, including *S. aureus* TISTR 517 and MRSA strain 142, MRSA strain 1096, MRSA strain 2468, *E. coli* TISTR 887, *K. pneumoniae* TISTR 1383, and *P. aeruginosa* TISTR 357. The FN33 isolate exhibited inhibitory activity against *S. aureus* TISTR 517 and MRSA strains 142, 1096, and 2468, with a particularly potent effect against MRSA strains (Table 1). However, the CFS of FN33 did not exhibit antibacterial activity against Gram-negative bacteria, suggesting selective antimicrobial activity against Gram-positive pathogens.

### 2.2. Production Kinetics of Antibacterial Components of FN33 Isolate

**Figure 1:**
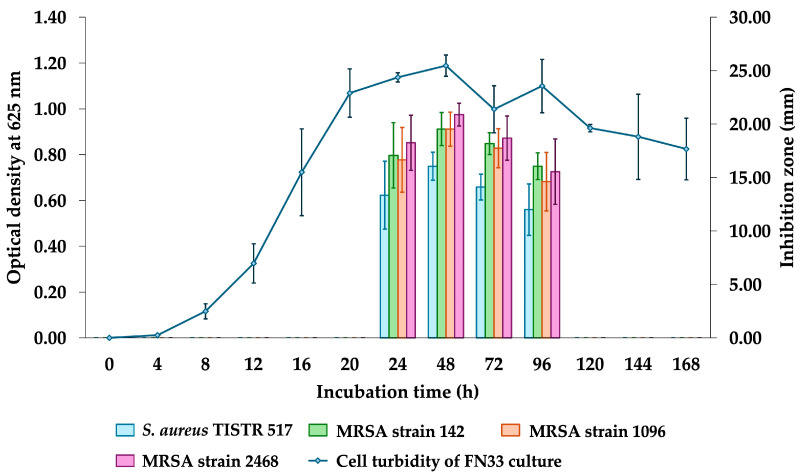
Growth curve and production kinetics of antibacterial components of FN33 isolate.

The antibacterial activity of the FN33 isolate was observed exclusively against Gram-positive bacteria. The production kinetics of antibacterial components in the CFS of the FN33 culture were investigated by measuring inhibition zones against *S. aureus* TISTR 517 and MRSA strains 142, 1096, and 2468 at different time intervals (Figure 1). At the initial phase of inoculation (within the first 4 h of incubation), the OD625 was maintained near zero, which indicated that the bacterial cells were undergoing adaptation. Between 8 and 24 h of incubation, the logarithmic phase was observed. OD625 was increased, which reflected active bacterial growth and metabolism. The inhibition zone was not observed during the first 0–20 h, indicating that no significant antibacterial component production had occurred. After 24 h of incubation, OD625 reached a plateau, indicating the stationary phase between 24 and 96 h. During this phase, inhibition zones were observed, suggesting that antibacterial components were actively secreted into the medium. The maximum inhibition zone was detected at 48 h, indicating peak antibacterial activity. A decline in inhibition zones was found after 48 h of incubation, before diminishing completely at 120 h. Statistical analysis was conducted to evaluate the antibacterial activity of FN33 against each tested strain. The results showed no significant difference in the inhibition zones among the MRSA strains across all incubation periods. However, a smaller inhibition zone was observed in *S. aureus* TISTR 517 at 48–72 h compared to the MRSA strains. FN33 demonstrated significantly higher antibacterial activity against the three MRSA strains than against *S. aureus* during the 48–72 h incubation period. The production kinetics suggested that 48 h of incubation was the optimal incubation time for FN33 culture to maximize the production of antibacterial activity against *S. aureus* TISTR 517 and MRSA strains.

### 2.3. Purification of the Antibacterial Components of FN33 Isolate

**Figure 2:**
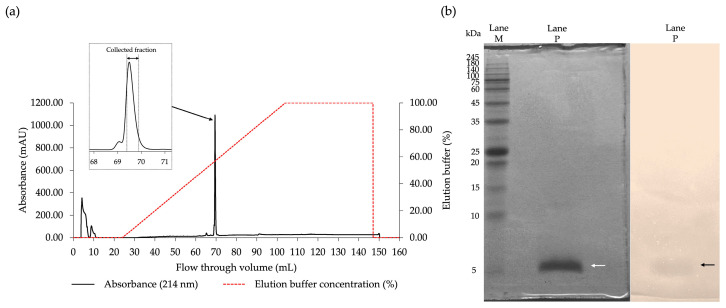
The purification of FN33 AMP was performed using reversed-phase chromatography. The inset area indicates the collected active fractions (**a**). The peptide band of the purified AMP (white arrow) was estimated for molecular weight by SDS-PAGE, compared to a protein marker (Lane M). The active protein band showed an inhibition zone (black arrow) as determined using soft agar overlay assay against MRSA strain 2468 (**b**).

The CFS of FN33 was subjected to protein precipitation using ammonium sulfate. Antibacterial activity against MRSA strain 2468 was detected in the precipitate obtained from the 25–75% ammonium sulfate saturation range. The maximum inhibition zone was observed at 50% of ammonium sulfate saturation. When the ammonium sulfate concentration was increased to 75%, the inhibition zone was reduced compared to that at 50% saturation. Therefore, the antibacterial component precipitated at 50% ammonium sulfate saturation was selected for the subsequent purification procedures. These included cation-exchange chromatography followed by reversed-phase chromatography. Antimicrobial activity in the eluent was detected in the fraction collected at a retention time of 17.50 min. This active fraction was further purified by reversed-phase chromatography. A single peak was found when the concentration of elution buffer reached 60% (Figure 2a). The collected fraction of reversed-phase chromatography was subjected to an investigation of the antimicrobial activity. The result confirmed that the antimicrobial fraction was retained in the purified fraction. The purification balance sheet (Table 2) was used to summarize the purification efficiency at each step. The total dried weight was progressively reduced after each purification step, which indicated the removal of other inactive components. Following 50% ammonium sulfate precipitation, the arbitrary activity increased, resulting in a higher specific activity (41.96 AU/mg) and a 4.02-fold purification factor. Subsequent cation-exchange chromatography further enriched the antibacterial substance from the FN33 fraction, yielding a specific activity of 125.71 AU/mg, corresponding to a 12.06-fold purification relative to the CFS. The purification factor increased stepwise, reaching 117.66-fold after reversed-phase chromatography, confirming the effective enrichment of active antibacterial substance. The highest specific activity (1226.67 AU/mg) was achieved in the final purified fraction, with a final yield of 5.27%, demonstrating the effectiveness of the purification process. The purified fraction was analyzed by SDS-PAGE to confirm its purity. The SDS-PAGE analysis revealed a single stained protein band in Lane P of the half-excised gel, corresponding to a molecular weight of approximately 5 kDa. A matching inhibition zone was observed in the other half-excised gel overlaid with MRSA strain 2468, confirming the antibacterial activity at the same location (Figure 2b). These results confirm that the stepwise purification procedures effectively isolated the antibacterial substance of FN33 while preserving its antibacterial properties, thereby making it suitable for further characterization.

### 2.4. De Novo Amino Acid Sequencing

**Figure 3:**
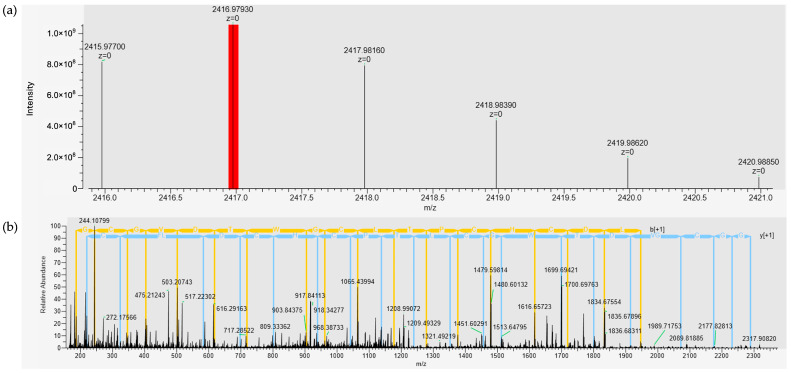
The full-scan spectra revealed mass at 806.32941 Da ([M+H]^+3^) and 1208.98962 Da ([M+H]^+2^) (**a**). The whole mass of peptide is shown before fragmentation of secondary mass spectrometer. MS/MS fragmentation patterns for the precursor ion 1208.98962 (**b**) with stepped NCE values of 38%.

The purified AMP was analyzed to determine its mass and structure by amino acid sequencing through de novo sequencing. The peptide sequence was subjected to full-scan spectra of mass. The analyzed peptide ions with charge state distributions generated through electrospray ionization (ESI) were measured in full-scan mode using Fourier transform mass spectrometry (FT-MS). The full-spectrum scanning at *m*/*z* 600–5000 revealed that the ion with the highest intensity was observed at *m*/*z* 806.32941 ([M+H]^+3^), followed by another significant ion at *m*/*z* 1208.98962 ([M+H]^+2^) (Figure S1a). Deconvolution of these spectra, focusing on the *m*/*z* ions at 806.32941 and 1208.98962, along with confirmation from the whole mass spectrum indicated a potential peptide mass of 2416.98 Da (Figure 3a). The stepped normalized collision energy (NCE) ranging from 28% to 38% was applied during MS/MS fragmentation for identifying the peptide sequence. This approach ensured comprehensive coverage of fragmentation patterns by generating both b– and y–ion series, which are crucial for de novo sequencing. The MS/MS fragmentation patterns for the precursor ion at *m*/*z* 806.32941 ([M+H]^+3^) and *m*/*z* 1208.98962 ([M+H]^+2^) were fragmented by various levels of collision energy. The fragmentation patterns for both the *m*/*z* of 806.32941 Da and 1208.98962 Da precursor ions were performed with the fragmentation at NCEs of 38% (Figure 3b and Figure S1b,c). The amino acid sequence of the FN33 peptide was NH_3_-1Glu-2Gly-3Gly-4Cys-5Gly-6Val-7Asp-8Thr-9Trp-10Gly-11Cys-12Leu-13Thr-14Pro-15Cys-16His-17Cys-18Asp-19Leu-20Phe-21Cys-22Thr-23Thr-COOH. The physicochemical properties of the peptide were predicted using ProtParam on the Expasy server. The analysis indicated a theoretical isoelectric point (pI) of 3.54 and a net negative charge of –3.2 at pH 7 (www.pepcalc.com). The grand average of hydropathy (GRAVY) score was 0.283. A slightly positive GRAVY value signifies a mild hydrophobic peptide [23]. The instability index of FN33 AMP was predicted to be 32.63. According to the instability index, proteins with values exceeding 40 are considered unstable in vivo [24]. The amino acid sequence similarity search in the antimicrobial peptide database (APD3 and DRAMP) revealed that FN33 AMP exhibited the highest similarity score of 42.86% to Hymo B, a plant defense peptide consisting of 25 amino acid residues. Hymo B is a cysteine-rich, cyclic peptide with a net charge of −1, originally isolated from *Hybanthus monopetalus* in Australia [25]. A sequence similarity search for the identified AMP was conducted using the UniProtKB/Swiss-Prot and AlphaFold databases, but no homologous sequences were identified.

### 2.5. Determination of Antibacterial Activity of FN33 AMP by Microdilution Assay

The microdilution assay was conducted to evaluate the antibacterial activity of FN33 AMP in comparison to standard antibiotics. The MIC for *S. aureus* TISTR 517 (16 µg/mL) was higher than that for the MRSA strains (8 µg/mL), indicating that the MRSA strains were more susceptible to FN33 AMP. The MBC values were 4-fold higher than the MIC for *S. aureus* TISTR 517 and 2-fold higher for the MRSA strains, suggesting that FN33 AMP exhibited a strong bactericidal effect against MRSA. Compared to vancomycin, which had an MIC and MBC of 2 µg/mL against both *S. aureus* TISTR 517 and the MRSA strains, FN33 AMP required a higher concentration to inhibit bacterial growth. In contrast, cefoxitin exhibited an MIC and MBC of 2 µg/mL against *S. aureus* TISTR 517; however, the MRSA strains were highly resistant, with the MIC and MBC values exceeding 64 µg/mL (Table 3).

### 2.6. Killing Kinetic Studies

**Figure 4:**
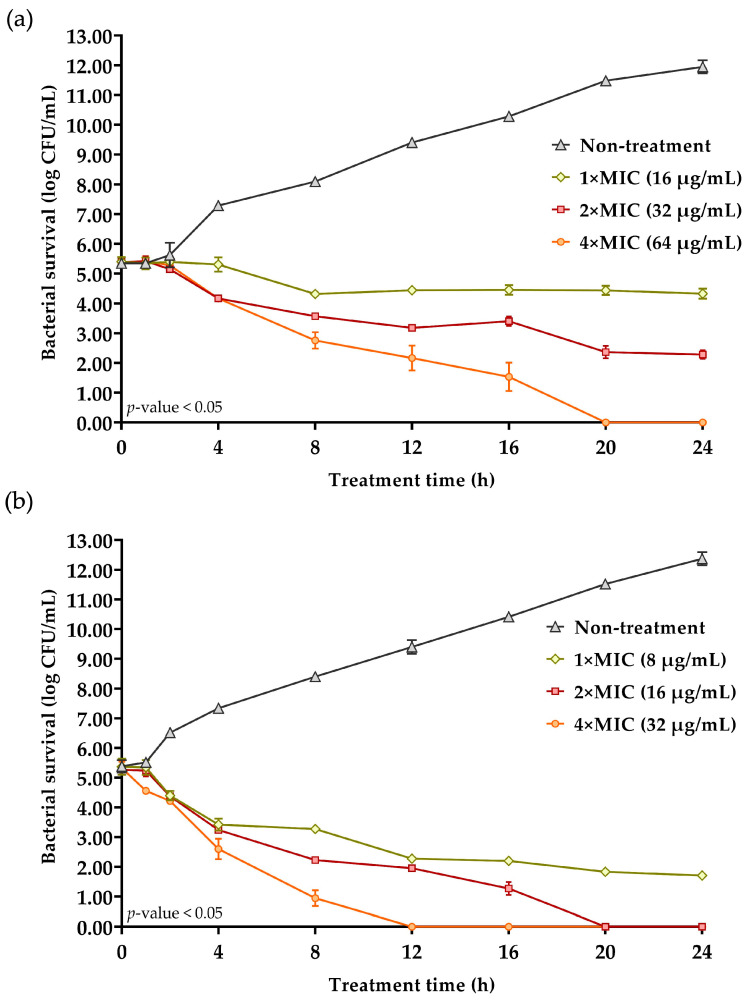
Time–kill curves of *S*. *aureus* TISTR 517 (**a**) and MRSA strain 2468 (**b**) treated with FN33 AMP at different concentrations. Bacterial viability was measured as log CFU/mL over time following treatment with 1× MIC (◇), 2× MIC (□), and 4× MIC (○), while the non-treatment (△) was included for comparison. Error bars represent the standard deviation of triplicate experiments.

The time–kill assay of FN33 AMP was conducted by measuring bacterial viability (log CFU/mL) at different time points. *S. aureus* TISTR 517 and MRSA strain 2468 were treated with FN33 AMP at 1×, 2×, and 4× MIC, while an untreated control was included for comparison. In the untreated group, bacterial growth increased over time, with a growth rate of 0.292 log CFU/h for *S. aureus* TISTR 517 and 0.2884 log CFU/h for MRSA strain 2468. At 1× MIC and 2× MIC, bacterial growth was inhibited by FN33 AMP, and reduction rates were measured as 0.0496 log CFU/h and 0.1322 log CFU/h, respectively. However, complete eradication of *S. aureus* TISTR 517 was not achieved at either concentration, even after 24 h of treatment. The result suggested that FN33 AMP exhibited a bacteriostatic effect at these concentrations. In contrast, FN33 AMP at 4× MIC exhibited antibacterial activity as demonstrated by a continuous decline in bacterial counts over time. The bacterial reduction rate at 4× MIC was 0.2704 log CFU/h. The bactericidal effect was confirmed by complete eradication within 20 h of treatment. In the 1× and 2× MIC groups, bacterial counts initially declined within the first 8 h but then stabilized. The killing rate against MRSA strain 2468 was faster than that observed for *S. aureus* TISTR 517 at all concentrations. A faster reduction rate of bacterial count was observed from 1× MIC to 4× MIC. At 1× MIC, the reduction rate was 0.1519 log CFU/h, but this concentration could not achieve an eradicative effect. In contrast, at 2× MIC and 4× MIC, the bacterial counts gradually decreased with reduction rates of 0.2498 log CFU/h and 0.4508 log CFU/h, respectively. The time to eradication at these concentrations showed no detectable bacterial counts within 20 h and 12 h of treatment, respectively. These findings indicate that FN33 AMP exhibits both concentration- and time-dependent bactericidal effects against MRSA at concentrations above 2× MIC. At 4× MIC, bacterial reduction continued until complete eradication at 12 h, whereas at 1× and 2× MIC, the *S. aureus* TISTR 517 counts stabilized after 8 h with no further significant reduction up to 24 h, confirming a bacteriostatic effect at lower concentrations. Notably, at 4× MIC, *S. aureus* TISTR 517 was less susceptible to FN33 AMP, as indicated by a lower reduction rate and a longer time required for complete bacterial eradication compared to MRSA strain 2468 (Figure 4).

### 2.7. Investigation of Bacterial Pathogens Treated with FN33 AMP by Scanning Electron Microscopy (SEM)

**Figure 5:**
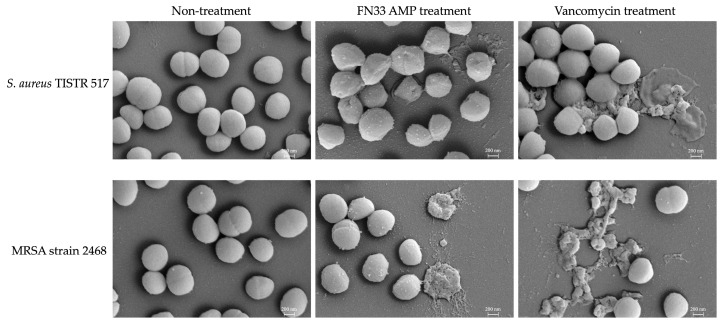
SEM images captured at 30,000× magnification. The morphological changes in *S. aureus* TISTR 517 and MRSA strain 2468 following treatment with 1× MIC of FN33 AMP and vancomycin for 18 h were observed. The non-treatment condition served as a negative control.

SEM was used to examine morphological alterations in the bacterial cells. *S. aureus* TISTR 517 and MRSA strain 2468 were treated with FN33 AMP and the standard antibiotic vancomycin at 1× MIC. Untreated *S. aureus* TISTR 517 and MRSA strain 2468 cells served as the control, exhibiting intact membranes with smooth and spherical morphology. This appearance reflected their natural cellular structure. After 16 h of FN33 AMP and vancomycin treatment, significant structural damage was observed, including cell rupture and lysis, indicating bacterial membrane disruption. The structural changes in the bacterial membrane following FN33 AMP treatment showed distinct differences between *S. aureus* TISTR 517 and MRSA strain 2468. The treated *S. aureus* exhibited a dented and rough surface with fewer lysed cells observed, whereas the treated MRSA showed a greater number of completely lysed cells. On the other hand, the treatment with vancomycin resulted in complete cell lysis in both bacterial strains (Figure 5). The SEM images of the treated cells showing lysis indicate that FN33 AMP possesses bacterial membrane-disrupting properties.

### 2.8. Assay for Bacterial Membrane Permeabilization

**Figure 6:**
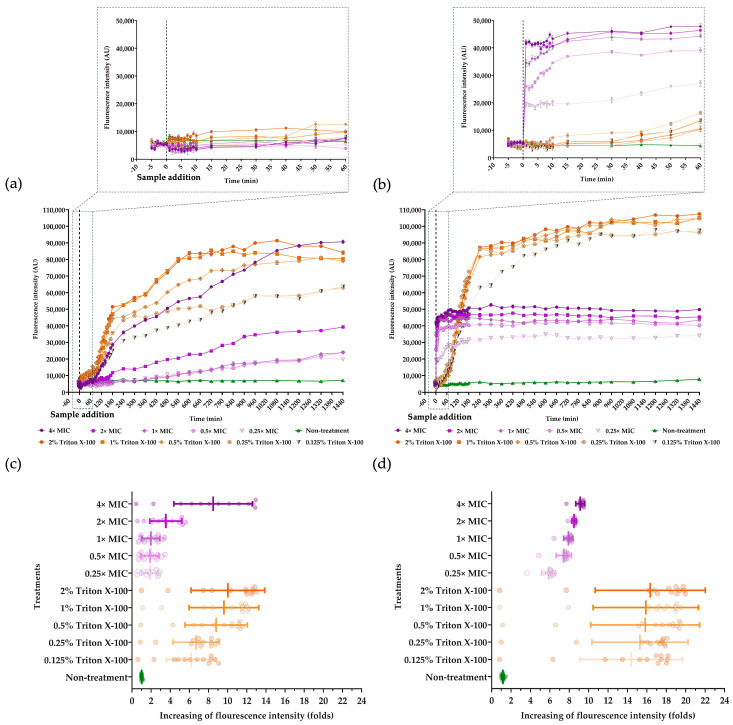
The Sytox Green uptake by FN33 AMP-treated bacterial cells represented the effect of FN33 AMP on cell permeability. The cell cultures of *S. aureus* TISTR 517 (**a**) and MRSA strain 2468 (**b**) were incubated at different concentrations of FN33 AMP (0.25×, 0.5×, 1×, 2×, and 4× MIC), while the various concentrations of Triton X-100 (0.125%, 0.25%, 0.5%, 1%, and 2%) were used as positive control to indicate the levels of cell membrane permeability. The membrane permeabilization of bacterial cells was determined by the increase in fluorescence intensity caused by the Sytox Green–DNA complex. The fluorescence intensity was observed over time for 24 h. The inset graphs show the expanded view of the fluorescence baseline before sample addition and the fluorescence intensity during the initial 60 min after sample addition. The increase in fluorescence intensity over the entire treatment period was expressed as fold changes, comparing different sample concentrations to the untreated condition of *S. aureus* TISTR 517 (**c**) and MRSA strain 2468 (**d**).

The Sytox Green uptake assay was conducted to assess membrane permeabilization associated with loss of membrane integrity. The fluorescence would be detected from a Sytox Green–DNA complex. The incubation with various concentrations of FN33 AMP (0.25×, 0.5×, 1×, 2×, and 4× MIC) was carried out to determine the effect on bacterial membrane permeability. Triton X-100 (0.125–2%) and an untreated sample were used as positive and negative controls, respectively. Fluorescence intensities were recorded 5 min before sample addition across all groups to ensure cell integrity prior to the experiment. A significant increase in fluorescence was detected immediately after the addition of FN33 AMP and Triton X-100, indicating membrane permeabilization in both *S. aureus* TISTR 517 and MRSA strain 2468. Notably, FN33 AMP produced a rapid increase in fluorescence intensity and a higher overall signal in MRSA strain 2468 than in *S. aureus* TISTR 517 at the moment of sample addition at 0.25×, 0.5×, 1×, and 2× MIC (Figure 6a,b, and their insets). In *S. aureus* TISTR 517, fluorescence intensities in the FN33 AMP-treated group were comparable to those in the positive control (Triton X-100) at the time of sample addition. After treatment, fluorescence intensity increased in a concentration- and time-dependent manner, indicating that membrane permeabilization was influenced by both the AMP concentration and treatment time. The average increase in fluorescence intensity was 1.88 ± 0.83-, 1.89 ± 0.91-, 1.98 ± 0.92-, 3.54 ± 1.64-, and 8.49 ± 4.31-fold higher than the untreated group for 0.25×, 0.5×, 1×, 2×, and 4× MIC of FN33 AMP, respectively (Figure 6a,b). For MRSA strain 2468, fluorescence intensity was higher than the positive control at the time of sample addition and remained concentration-dependent. The average fluorescence increase was 5.87 ± 0.72-, 7.44 ± 0.77-, 7.91 ± 0.48-, 8.49 ± 0.25-, and 9.13 ± 0.46-fold higher than the untreated group for 0.25×, 0.5×, 1×, 2×, and 4× MIC of FN33 AMP, respectively. These results indicate that the membrane of MRSA strain 2468 is more susceptible to permeabilization by FN33 AMP than that of *S. aureus* TISTR 517 (Figure 6c,d). However, in *S. aureus*, a level of membrane permeabilization exceeding that of MRSA strain 2468 was only observed when the concentration was increased to 4× MIC. Membrane permeabilization caused by Triton X-100 was distinct from that induced by FN33 AMP in both *S. aureus* TISTR 517 and MRSA strain 2468. In *S. aureus* TISTR 517, 0.5% Triton X-100 induced fluorescence levels comparable to those observed with FN33 at 4× MIC. In contrast, in MRSA strain 2468, fluorescence at 4× MIC of FN33 AMP reached only 60% of the intensity observed with 0.5% Triton X-100, suggesting that the AMP’s mode of action differs from detergent-induced permeabilization.

### 2.9. Stability Studies of FN33 AMP

The stability of the AMP was evaluated by assessing its activity retention against MRSA strain 2468 under various conditions. Under control conditions, the AMP remained fully stable over 12 h. The preserved activity at 30 °C and 40 °C was comparable to the control condition. However, the activity gradually declined at 60 °C, 80 °C, and 100 °C, showing a significant time-dependent reduction in activity (*p*-value < 0.05). Overall, up to 20% of activity was lost over 12 h due to elevated temperatures. The loss of activity became more marked when the AMP was autoclaved at 121 °C and 15 psi for 15 and 30 min. These results indicate that temperature affects the antibacterial activity in a time- and temperature-dependent manner.

Protease exposure significantly affected FN33 AMP stability. Incubation with proteinase K and α-chymotrypsin led to a complete loss of antimicrobial activity at all tested time points, indicating that FN33 AMP is highly susceptible to degradation by these enzymes (*p*-value < 0.05). A noticeable decline occurred after prolonged exposure, suggesting progressive cleavage rather than immediate inactivation. In contrast, trypsin digestion resulted in stable activity over time (Table 4). The digestion of protease enzymes against FN33 AMP confirmed its proteinaceous nature.

The antibacterial activity of FN33 AMP varied depending on the surfactant combination. The cationic surfactant, cetyltrimethylammonium bromide (CTAB), caused a slight reduction in activity that indicated minimal interference against FN33 AMP. Moreover, the non-ionic surfactant, Triton X-100, significantly reduced the antibacterial activity of FN33 AMP. In contrast, the anionic surfactant, sodium dodecyl sulfate (SDS), enhanced its activity. The use of surfactants with different charge properties influenced the antibacterial activity of FN33 AMP.

FN33 AMP exhibited reduced activity under extreme pH levels. Strongly acidic (pH 1) and strongly alkaline conditions (pH 10 and 14) impaired the antibacterial activity of FN33 AMP. Although the peptide retained higher stability at pH 4, its activity was still lower than the control, indicating some impact of mild acidity. In contrast, the stability of FN33 AMP was well maintained at pH 8.

### 2.10. Determination of Peptide Secondary Structure by Molecular Dynamics (MD) Simulation

**Figure 7:**
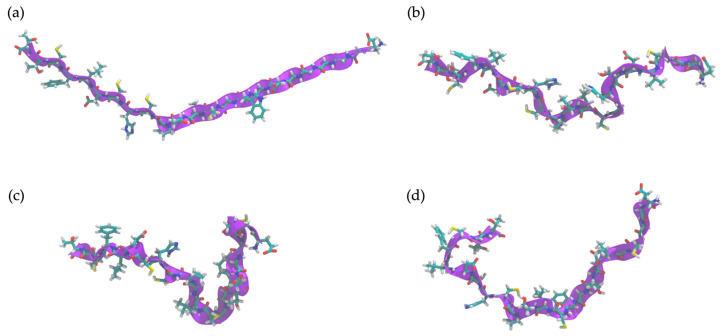
Snapshots illustrating the time evolution of FN33 AMP’s structural dynamics in water. Atomistic MD simulations of FN33 AMP at different time points: 0 ns (**a**), 1 ns (**b**), 10 ns (**c**), and 100 ns (**d**), showing structural transitions over the simulation period. FN33 AMP was modeled using ribbon and licorice to visualize the peptide backbone and side chains, respectively, with atoms colored according to the Corey–Pauling–Koltun scheme.

**Figure 8:**
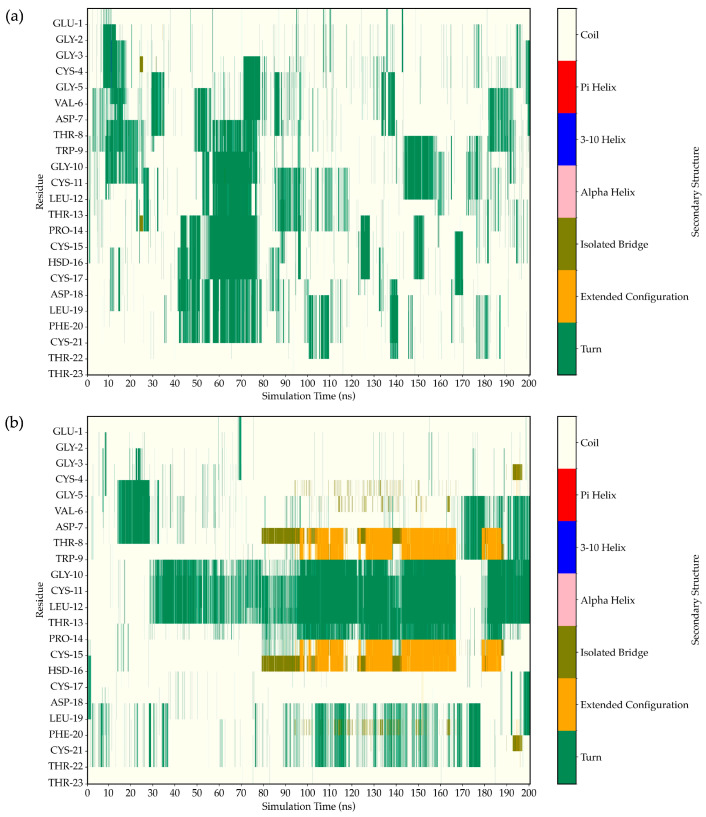
Time evolution of FN33 AMP’s secondary structure during MD simulation in water (**a**) and in the presence of a bacterial membrane (**b**). The *Y*-axis represents amino acid residue positions, and the *X*-axis denotes simulation time (ns). This visualization tracks structural transitions over time, highlighting conformational stability and dynamic folding behavior.

The time evolution of FN33 AMP’s structural dynamics was investigated in both aqueous and *S. aureus* membrane environments. The simulation result indicated a high flexibility of the peptide structure in an aqueous environment throughout the 0–100 ns simulation (Figure 7a–d). In aqueous conditions, FN33 AMP predominantly remained in a random coil and turn conformation, lacking stable secondary structure formation such as β-sheets or α-helices (Figure 8a). In contrast, membrane interaction significantly influenced FN33 AMP’s secondary structure dynamics. During the 0–80 ns timeframe, the peptide maintained a random coil and turn, similar to its behavior in an aqueous environment. However, distinct structural changes were observed. The formation of a stable isolated bridge and extended configurations indicated that the β-sheet structures in an anti-parallel orientation were contributed by amino acid residues Cys-15–His-16 and Thr-8–Trp-9 (Figure 8b). The findings suggest that membrane interaction induces a disorder-to-order transition, contributing to the stabilization of the peptide structure.

### 2.11. In Silico Study of AMP Dynamics in a Bacterial Membrane Model

**Figure 9:**
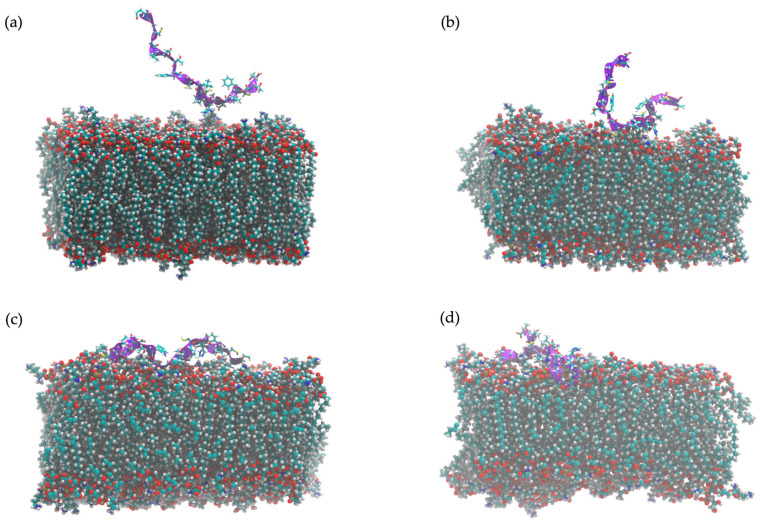
Time evolution of FN33 AMP dynamics within a bacterial membrane model composed of 1,2-O-dipalmitoyl-sn-glycero-3-phospho-(1′-rac-glycerol) (DPPG) (40%), 1,2-O-dipalmitoyl-sn-glycero-3-phospho-rac-(3-lysyl(1′-glycerol)) (Lysyl-DPPG) (52%), and 1,1′,2,2′-tetramyristoyl cardiolipin (TMCL) (8%). Snapshots illustrate FN33 AMP interaction at different simulation time points: initial state (0 ns) (**a**), early interaction (1 ns) (**b**), membrane association phase (10 ns) (**c**), and stabilized β-sheet conformation (100 ns) (**d**). Atomic colors are represented using the Corey–Pauling–Koltun color scheme.

**Figure 10:**
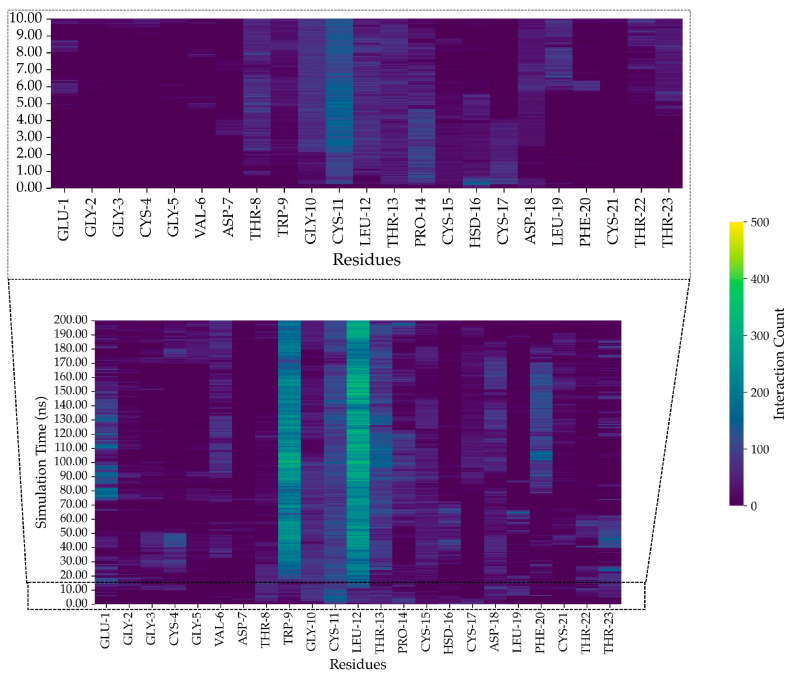
Heat map representation of FN33 AMP interactions with the bacterial membrane over a 0–200 ns simulation period. The inset illustrates the initial binding event between the AMP and the bacterial membrane within the first 10 ns.

The MD simulations employed an *S. aureus* bacterial membrane model, consisting of 1,2-O-dipalmitoyl-sn-glycero-3-phospho-(1′-rac-glycerol) (DPPG) (40%), 1,2-O-dipalmitoyl-sn-glycero-3-phospho-rac-(3-lysyl(1′-glycerol)) (Lysyl-DPPG) (52%), and 1,1′,2,2′-tetramyristoyl cardiolipin (TMCL) (8%) [26]. The snapshots of FN33 AMP dynamics within the bacterial membrane model provide evidence of its structural transitions. Over the total simulation time of 200 ns, the peptide reached its final configuration by successfully inserting into the bacterial membrane (Figure 9a–d). This structural transition suggests a dynamic rearrangement, indicating the interaction between peptide and membrane. The strong peptide–lipid interactions at Trp-9 and Leu-12 underscore the importance of these residues in membrane anchoring, which is a key aspect of FN33 AMP’s antibacterial mechanism. At the initial stage of the simulation (0 ns), FN33 AMP was positioned parallel to the lipid bilayer surface (Figure 9a). Within 1 ns, the peptide rapidly moved toward the membrane, while maintaining its parallel orientation (Figure 9b). By 10 ns, the entire peptide structure was lying on the membrane surface (Figure 9c). Finally, at 100 ns, the formation of β-sheets was observed involving Cys-15–His-16 and Thr-8–Trp-9 residue pairings (Figure 9d). The heat map illustrates the intensity of interactions between the amino acid residues of FN33 AMP and the lipid components at different time points during the MD simulations. The interaction between FN33 AMP and the simulated membrane was observed within 1 ns of the simulations, with His-16 and Asp-18 initially binding to the membrane surface (Figure 10, inset). Subsequently, heat map analysis revealed a high interaction intensity at Cys-11. At this stage, the peptide adopted a parallel orientation relative to the bilayer surface, indicating a conformational shift in response to membrane binding. Between 20 ns and 200 ns, the interaction intensity was highest at Trp-9 and Leu-12, suggesting that these residues played a crucial role in membrane embedding (Figure 10). The aromatic ring of tryptophan facilitated aromatic stacking interactions, which helped stabilize the peptide–membrane interface. Similarly, the hydrophobic side chain of leucine contributed to membrane insertion through hydrophobic interactions.

**Figure 11:**
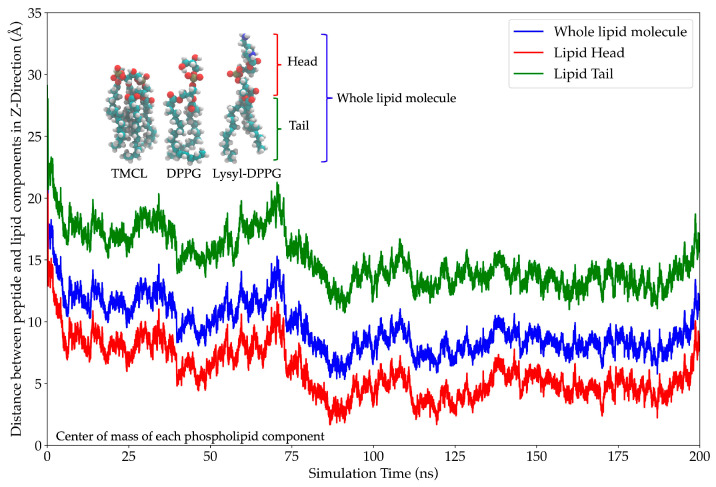
Time evolution of the center-of-mass (COM) distance between FN33 AMP and lipid components during MD simulations. The *Y*-axis represents the COM distance (Å) between FN33 AMP and lipid components. The *X*-axis represents simulation time (ns). The blue, red, and green lines correspond to the whole lipid molecule, lipid headgroup, and lipid tail region, respectively.

The center-of-mass (COM) analysis was used to evaluate the peptide’s anchoring across the membrane over time, providing insights into its binding dynamics and insertion depth [27]. The results revealed a time-dependent association between the AMP and the lipid components. At the start of the simulation (0 ns), the peptide was positioned 20 Å away from the lipid head, indicating a lack of interaction with the membrane surface. The distance between the peptide and various lipid components (whole lipid molecule, lipid head, and lipid tail) decreased rapidly within the first 5 ns and remained stable until approximately 70 ns. This stage indicated a parallel orientation of the peptide lying on the membrane surface. Beyond 70 ns, the COM distance initially increased and then continued to decrease, which reflected a change in the peptide’s COM due to β-sheet formation. The overall reduction in COM across all lipid regions suggested progressive membrane association. By 90 ns, the peptide reached its deepest penetration and achieved a minimum COM distance of nearly 0 Å at the lipid head layer. The peptide’s interaction with lipid headgroups containing glycerol, phosphate, and lysyl groups underscores the importance of electrostatic and polar interactions in membrane binding. These interactions indicate strong membrane association and potential surface insertion (Figure 11).

### 2.12. Effect of AMP on Albumin Denaturation and 50% Inhibitory Concentration (IC_50_) of DPPH

The anti-inflammatory and antioxidant activities of FN33 AMP are considered crucial for the development of a drug with both antibacterial and immunomodulatory properties for the treatment of bacterial infections. FN33 AMP demonstrated efficacy in protecting albumin from denaturation. The albumin protective effect of the AMP was greater than that of the positive control group (diclofenac sodium). Moreover, at a concentration of 500 µg/mL, the AMP provided significantly greater protection than all tested doses of diclofenac sodium (*p*-value < 0.05). The albumin protection effect was shown to be dose-dependent. The protective potency of FN33 AMP at 250 µg/mL of FN33 was comparable to that of 500 µg/mL of diclofenac (Table 5). The DPPH radical scavenging assay was used to assess the antioxidative efficacy of the AMP. The half-maximal inhibitory concentration (IC_50_) of the AMP was 11.66 ± 2.87 µg/mL, while that of ascorbic acid was 6.78 ± 1.58 µg/mL. FN33 AMP exhibited lower DPPH-scavenging activity than ascorbic acid (Table 6).

### 2.13. Sequencing of FN33 Genome

**Figure 12:**
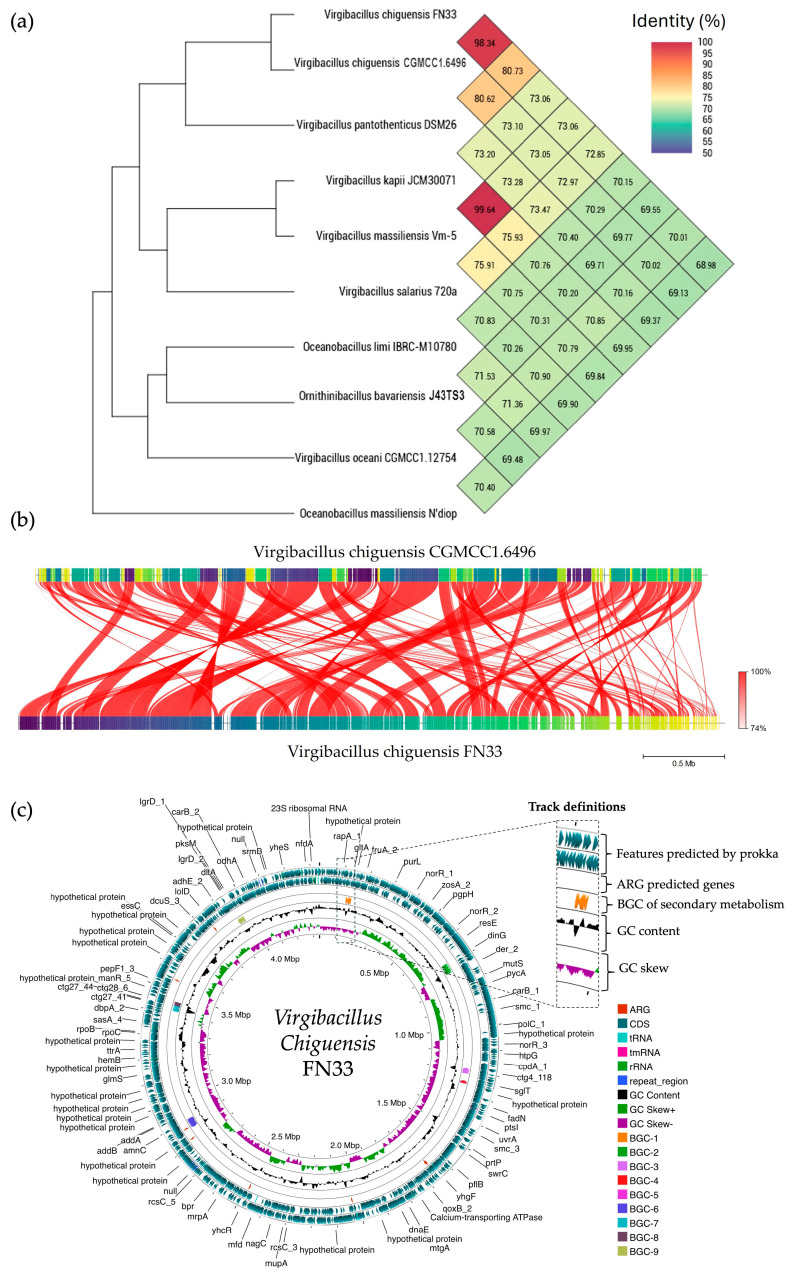
The taxonomic relationship of FN33 to other *Virgibacillus* species was analyzed using phylogenetic tree construction and genome similarity assessments, which were performed using the TYGS database and OrthoANI (**a**). The ANI was computed between *Virgibacillus chiguensis* CGMCC1.6496 and *Virgibacillus chiguensis* FN33. FastANI was used to visualize orthologous sequence mappings. Red line segments represent orthologous regions identified by FastANI for ANI estimation (**b**). The genomic features of *Virgibacillus chiguensis* FN33 are displayed in a circular genome map generated using Proksee. Genome map is visualized with separate tracks, indicating different categories of genomic information (**c**).

A single colony of FN33 exhibited a circular, flat, translucent, and white morphology with an irregular margin. Microscopic observation revealed rod-shaped, endospore-forming, and Gram-positive bacteria. Species identification was conducted based on whole-genome sequence analysis. The sequencing reads were of high quality and the assembled genome had a size of 4,336,377 bp with a sequencing depth of 394×, which was constructed through de novo assembly using the SPAdes method. The GC content was 36.50%. The genome showed a completeness of 99.33% and a contamination level of 0.22%. The genome sequence of *Virgibacillus chiguensis* FN33 was deposited in the National Center of Biotechnology Information (NCBI) database under the accession number JBFRBO000000000. The taxonomy of FN33 was predicted using genome-based sequencing and analyzed through the Type (Strain) Genome Server (TYGS) and Orthologous Average Nucleotide Identity (OrthoANI). Within the genus *Virgibacillus*, OrthoANI identity values ranged from 70.01% to 98.34%. Among these, FN33 showed the closest relation to *Virgibacillus chiguensis* CGMCC1.6496 with an identity of 98.34% (Figure 12a). FastANI tool version 1.1.0 was used to visualize orthologous DNA sequence mappings between *Virgibacillus chiguensis* CGMCC1.6496 and *Virgibacillus chiguensis* FN33. The FastANI result showed 98.28% similarity across the entire orthologous genome mapping, indicating that highly similar DNA sequences are distributed throughout the reference genome (Figure 12b) [28,29,30]. The pairwise comparison was calculated using the Genome BLAST Distance Phylogeny (GBDP) method to confirm the taxonomic relationship of FN33 against genomes in the TYGS database (https://tygs.dsmz.de, accessed on 19 July 2024). The GBDP results showed similarity scores of 84.7% (95% CI, 80.9–87.8%), 85.2% (95% CI, 82.5–87.6%), and 87.7% (95% CI, 84.7–90.2%) for distance formulas *d_0_*, *d_4_*, and *d_6_*, respectively. The GC content of FN33 was identical at 36.50% to that of the type strain *Virgibacillus chiguensis* CGMCC1.6496, further supporting the classification. These results indicated that the FN33 strain has the closest taxonomic relationship to *Virgibacillus chiguensis* CGMCC1.6496 at the species level. The protein coding sequences (CDS) were annotated by Rapid Prokaryotic Genome Annotation (Prokka) version 1.14.6, including biosynthetic gene clusters (BGCs) of secondary metabolites, which were annotated by Antibiotics and Secondary Metabolite Analysis Shell (antiSMASH) version 7.0. The prediction of antimicrobial-resistant genes localized in the genome were annotated using the Comprehensive Antibiotic Resistance Database (CARD). The annotation systems were visualized using Proksee. The analyzed genome was presented with the annotations by a circular map with a number of different functions of genes that were classified and represented using various colors (Figure 12c). The 4085 features were annotated using Prokka, and there were 4010 CDSs, seven rRNAs, one tmRNA, 61 tRNAs, and six CRISPRs in the genome. The BGCs of the secondary metabolites as antimicrobial compounds were counted by antiSMASH, which provided nine BGCs with different similarities based on orthologs in the databases. The predicted BGCs of the secondary metabolites revealed involvement in the biosynthesis of three class II lanthipeptides, three type III polyketide synthases (PKSs), one ribosomally synthesized and post-translationally modified peptide (RiPP), one lassopeptide, and one non-ribosomal peptide synthetase (NRPS). Antimicrobial resistance (AMR) genes were predicted using the CARD database. Glycopeptide resistance genes were identified with sequence identities ranging from 33.7% to 49.66%, aligning with 52.25% to 126.12% of the reference sequences. Genes associated with antibiotic efflux showed sequence identities between 40.95% and 59.40%, covering 100.00% to 116.82% of the reference sequences. Phosphonic antibiotic resistance genes were predicted with 60.14% identity, corresponding to 100.72% coverage of the reference sequence.

## 3. Discussion

Antimicrobial resistance (AMR) poses a significant global health challenge. MRSA has rapidly become the most prevalent resistant pathogen detected across various regions, including Europe, the United States, North Africa, the Middle East, and East Asia. MRSA is categorized into hospital-acquired MRSA and community-acquired MRSA [31]. Consequently, there is a pressing need to discover new antimicrobial agents. This resistance of MRSA is attributed to the presence of the mecA or mecC gene, which encodes penicillin-binding protein 2a (PBP2a), a protein that has low affinity for semi-synthetic penicillins, thereby rendering these antibiotics ineffective [32]. In this study, cefoxitin was used to determine the susceptibility profile of penicillin-class antibiotics and served as a surrogate marker for oxacillin and methicillin resistance in *S. aureus*. All three MRSA strains tested were resistant to cefoxitin but remained sensitive to both vancomycin and FN33 AMP. These findings highlight the potential effectiveness of the newly discovered AMP in targeting methicillin-resistant *S. aureus*.

Information on the characteristics of bacteriocin-producing *Virgibacillus* is quite limited. There have been reports of AMPs from *Virgibacillus* species. Virgicin, an AMP from *Virgibacillus* sp. strain AK90, exhibits inhibitory activity against Gram-positive bacteria and inhibits biofilm formation by *Enterococcus faecalis* [33]. AntiSMASH analysis of the *Virgibacillus chiguensis* FN33 genome sequence revealed the presence of a putative bacteriocin biosynthetic cluster containing the essential genes for lanthipeptide synthesis [33]. The genomic information of FN33 supports the identification of the AMP as a class II lanthipeptide, based on the presence of multiple cysteine residues. The similarity of the lanthipeptide structure to FN33 AMP was found in five reviewed lantipeptides from the UniProtKB database, including lantibiotic 107,891 (P85065), mutacin (P80666), epdermin (P08136), and gallidermin (P21838), found from *Microbispora* sp. strain 107891, *Streptococcus mutans* strain Ny266, *Staphylococcus epidermidis,* and *Staphylococcus gallinarum* (F16/P57) Tü3928, respectively [34,35,36]. However, confirmation of the biosynthetic gene cluster (BGC) responsible for FN33 AMP production will require further validation through gene knockout or gene expression studies [37]. Antimicrobial resistance genes, such as those encoding transporters or efflux pumps, are commonly found in *Virgibacillus chiguensis* FN33 and other AMP-producing bacteria, as they play a crucial role in exporting synthesized AMP molecules outside the cell [38].

Microorganisms produce bacteriocins as a competitive strategy to secure resources in microbial communities. However, in the early stages of incubation, when nutrients were abundant, bacteriocin production may have been suppressed until resource competition intensified [39]. The antibacterial activity of FN33 CFS was observed starting at 24 h after culturing, and it was found that the antibacterial substances showed the highest production of antibacterial activity at 48 h during the stationary phase. The purified antimicrobial peptide was identified with the sequence NH_3_–Glu–Gly–Gly–Cys–Gly–Val–Asp–Thr–Trp–Gly–Cys–Leu–Thr–Pro–Cys–His–Cys–Asp–Leu–Phe–Cys–Thr–Thr–COOH, and it was found to possess membrane-disruptive properties.

The Sytox Green uptake assay was used to determine the bacterial killing mechanism. The cationic dye can only penetrate compromised membranes and binds to intracellular DNA, resulting in a significant increase in fluorescence intensity, which indicates a loss of cytoplasmic membrane integrity [40]. FN33 AMP exhibited stronger cell membrane permeabilization in MRSA strain 2468 compared to *S. aureus* TISTR 517. These findings were consistent with agar well diffusion assays and production kinetic studies. These results showed that the MRSA strain 2468 was more susceptible to the antibacterial substance from FN33 than *S. aureus* TISTR 517. A previous study reported varying MIC values of an AMP against methicillin-susceptible *S. aureus* (MSSA) and MRSA. The MIC was 250 µg/mL for MSSA strain SA1911&1911B and 125 µg/mL for MRSA ATCC 35139 [41]. In another study, the MIC was 64 µg/mL for MSSA strain RN4420 and 32 µg/mL for MRSA strain MW2 [42]. Under SEM, the morphological changes induced by FN33 AMP and vancomycin will cause cell rupture and lysis, but the degree of membrane damage may differ between the two treatments. Furthermore, the degree of cell rupture and lysis correlated with the MIC and MBC values of FN33 AMP and vancomycin. The faster killing rate of FN33 AMP in MRSA suggested greater susceptibility of drug-resistant strains, possibly due to differences in membrane composition or interaction with the peptide. In *S. aureus*, key membrane lipids include DPPG, Lysyl-DPPG, and cardiolipin [43]. It has been reported that the lipid composition of vancomycin-resistant MRSA differs from that of *S. aureus*, with higher levels of Lysyl-DPPG in MRSA [26]. Elevated Lysyl-DPPG levels have been associated with increased resistance to cationic AMPs and other positively charged antibacterial agents [44]. Understanding how lipid composition and whole-cell charge contribute to the increased susceptibility of MRSA compared to *S. aureus*, particularly through alterations in membrane structure and fluidity, warrants further investigation [45].

Despite the vast diversity in their primary structures, most documented AMPs share a common characteristic: a high content of cationic and hydrophobic amino acids. These amino acids are arranged in specific regions of the molecule, contributing to the peptide’s functional properties [17]. The amino acid sequence of FN33 AMP contains one glutamic acid residue and two aspartic acid residues and does not include arginine and lysine; therefore, it is characterized as an anionic AMP. Only *S. aureus*, including the MSSA and MRSA strains, showed susceptibility to FN33 AMP. However, it exhibited no antimicrobial activity against Gram-negative bacteria. Many AMPs possess cationic properties, allowing them to interact with the bacterial cytoplasmic membrane through both electrostatic and hydrophobic forces. The membrane is typically composed of negatively charged phospholipids. However, *S. aureus* has evolved various mechanisms to diminish the efficacy of cationic AMPs. These include neutralizing the negative charge of the bacterial cell membrane by covalently modifying anionic components such as teichoic acids, phospholipids, and lipid A, using energy-dependent pumps to expel cationic AMPs, adjusting membrane fluidity, and degrading cationic AMPs with proteases [17]. *S. aureus* possesses the positively charged lipid Lysyl-DPPG at approximately 40–50% [43]. The anionic FN33 AMP is likely to strongly interact with this lipid, thereby associating with the headgroup region of the *S. aureus* membrane. It is believed that anionic AMPs could be useful in treatment, especially in cases where bacteria have become resistant to cationic AMPs [20].

The stability studies of FN33 AMP exhibited significant susceptibility to proteinase K and α-chymotrypsin, while showing resistance to trypsin digestion. FN33 AMP contains Trp-9, which is an aromatic amino acid that can be cleaved by proteinase K and α-chymotrypsin. In contrast, its resistance to trypsin digestion is due to the absence of lysine residue in the peptide structure [46]. The stability profile suggests potential limitations for therapeutic applications in protease-containing environments. This underscores the need for structural modifications or formulation strategies to improve its resistance to enzymatic degradation. Furthermore, the findings highlight the importance of surfactant compatibility when incorporating FN33 AMP into practical formulations, ensuring its stability and effectiveness in diverse applications. The antibacterial effectiveness of FN33 AMP was influenced by the type of surfactant applied. A higher antibacterial effect was observed in the presence of the anionic surfactant SDS, whereas the cationic surfactant CTAB and the nonionic surfactant Triton X-100 led to a reduction in AMP activity. The strong electrostatic interactions and hydrophobicity may compromise its antimicrobial function. However, careful consideration is necessary to optimize compatibility. Understanding the interplay between the AMP and surfactants is crucial for designing pharmaceutical products where surfactants are commonly used. The finding was consistent with another study that focused on a cationic AMP [47]. According to previous research, surfactant–protein interactions primarily occur through electrostatic forces and hydrophobic associations. Electrostatic interactions arise from the attraction or repulsion between charged surfactant headgroups and specific charged regions on peptides. Additionally, research suggests that while the charge of the surfactant headgroup influences these interactions, hydrophobic forces from surfactant chains play a crucial role in binding stability [48,49]. The cationic surfactant likely interacts electrostatically with the anionic FN33 AMP, reducing its overall charge and consequently diminishing its ability to interact with the headgroups of DPPG, Lysyl-DPPG, and cardiolipin. As a result, the weakened charge-based interaction between the AMP and lipid components correlates with a reduction in its antibacterial activity. In contrast, the anionic surfactant SDS enhanced antibacterial activity, possibly by increasing the ionic nature of the anionic AMP, thereby strengthening its interaction with bacterial membranes. On the other hand, Triton X-100 may have reduced the antibacterial activity due to hydrophobic interactions with the AMP, potentially altering its structural conformation. However, to fully elucidate these mechanisms, further investigation into the structural changes in FN33 AMP following exposure to different surfactants is recommended.

MD simulations offer in-depth analysis and provide the insights into bacterial membrane structure and dynamics. This approach has proven to be a powerful tool for uncovering key features of membrane architecture and organization, aiding in the investigation of AMP mechanisms and secondary structure behavior within the bacterial membrane [50]. The ability to form secondary structures is a key factor influencing the antimicrobial activity of AMPs. Upon interacting with bacterial membranes, AMPs undergo structural rearrangement, adopting a stable secondary conformation [51]. By 200 ns of MD simulations, FN33 AMP is inserted into the membrane, with Trp-9 and Leu-12 playing key roles in anchoring through aromatic stacking and hydrophobic interactions. The β-sheet structured AMPs such as β-defensins have been reported to contain cysteine-rich sequences that form hairpin-like structures. These peptides use their β-sheet conformation along with amphipathic properties to penetrate and disrupt bacterial cell membranes [52]. The membrane-disruptive properties of AMPs are commonly associated with electrostatic interactions between their positive net charge and the negatively charged components of bacterial membranes. In contrast, anionic AMPs have rarely been reported. Anionic AMPs typically carry a net charge between -1 and -8 and interact with positively charged components of bacterial membranes. An example is maximin H5, which employs a carpet-like model involving interactions between aspartic acid residues in the peptide structure and the membrane of *S. aureus*, leading to membrane disruption and cell death [53]. This supports the membrane interaction mechanism of FN33 AMP, which involves initial binding through aspartic acid and histidine residues, followed by dominant interactions mediated by hydrophobic amino acids during the later stages.

This study also evaluated the anti-inflammatory activity of FN33 AMP by assessing its ability to prevent heat-induced albumin denaturation, as inflammation plays a crucial role in bacterial infections. This multifunctional profile supports its potential as a therapeutic agent for treating infections associated with inflammation and oxidative stress and provides a promising foundation for future product development [54,55]. However, further investigation is required to confirm these preliminary observations, including cellular assays, mechanistic studies, and in vivo evaluations to establish the therapeutic relevance and safety of FN33 AMP in a physiological context.

Further studies are needed to investigate the molecular-level mechanisms of FN33 AMP and to identify the essential amino acid residues involved in its interaction and contribution to antibacterial activity through peptide structure modification. The druggability of the AMP should also be evaluated by assessing its pharmacokinetic and pharmacodynamic profiles as well as its clinical safety through in vivo and clinical studies. The peptide’s stability under elevated temperatures and protease-rich environments is a critical concern, underscoring the need for structural modifications to ensure effective delivery to the site of infection. Integrating genomic insights with rational peptide design may support the optimization of AMP production, particularly within the framework of synthetic biology for pharmaceutical applications. The findings from this study provide fundamental knowledge that could initiate the exploration and utilization of marine bacteria through bio-inspiration for the development of novel drug candidates, especially in response to the growing challenge of antimicrobial resistance.

## 4. Materials and Methods

### 4.1. Sample Collection and Bacterial Isolation

Mangrove sediment samples were collected from a depth of 10–15 cm at Nakhon Si Thammarat, Thailand. They were stored in clean polyethylene bags and transported in an ice box. Ten grams of sediment was placed into a sterile flask and diluted with 90 mL of sterile 0.85% NaCl solution (RCI Labscan Ltd., Bangkok, Thailand). The mixture was then agitated at 150 rpm for 30 min in a shaking incubator and subsequently heated at 60 °C for 30 min to facilitate spore selection. Serial dilutions were made up to 10^−6^, and 100 µL of each dilution was spread onto Mueller–Hinton (MH) agar, Zobell Marine (ZM) agar, and 1.5% NaCl-supplemented Starch Casein (SC) agar (Titan Biotech Ltd., Rajasthan, India). The plates were incubated at 30 °C for 7 days to allow colony growth before streaking to obtain pure isolates [56].

### 4.2. Screening of Antibacterial Activity Using the Soft Agar Overlay Method Against MRSA Strain

A single colony of the pure isolate was spotted onto solid media prepared during the bacterial isolation procedure and incubated at 30 °C for 3 days. MRSA strain 2468 was used as the test pathogen and subcultured on MH agar at 37 °C for 18 h. The MRSA suspension was prepared by transferring a colony from the subculture into 0.85% sterile NaCl solution. The turbidity of the suspension was adjusted to an optical density (OD) of 0.1 at 625 nm (Genesys 20, Thermo Scientific, Waltham, MA, USA). A 1 mL aliquot of OD-adjusted MRSA suspension (1 × 10^8^ CFU/mL) was mixed with 9 mL of molten 0.7% agar containing MH medium and overlaid onto plates previously seeded with bacterial isolates. The overlaid plates were incubated at 37 °C for 24 h. Antimicrobial activity was evaluated by observing the formation of an inhibition zone around the spotted colonies [57].

### 4.3. Verification of Antibacterial Activity by Agar Well Diffusion Method

The isolate that exhibited an inhibition zone against MRSA strain 2468 in the soft agar overlay assay was precultured for 24 h, then used to prepare a cell suspension in 0.85% sterile NaCl solution to serve as the starter culture. The optical density of the starter cultures was adjusted to 0.1 OD at 625 nm using 0.85% sterile NaCl solution before transferring 1 mL of the culture into 49 mL of fresh broth. The inoculum was then incubated at 30 °C with shaking at 150 rpm for 3 days. The CFS was obtained by centrifugation at 10,000× *g* at 4 °C for 15 min and filtered through a 0.2 µm sterile cellulose acetate syringe filter (Sigma-Aldrich, Warren, MI, USA). The antibacterial activity of the isolates was tested against *S. aureus* TISTR 517, *E. coli* TISTR 887, *K. pneumoniae* TISTR 1383, and *P. aeruginosa* TISTR 357, obtained from the Thailand Institute of Scientific and Technological Research (TISTR), Thailand, and three MRSA strains (strains 142, 1096, and 2468), provided by the medical technology laboratory of the School of Allied Health Sciences, Walailak University, Thailand. The pathogenic bacteria were incubated on MH agar at 37 °C for 18 h to obtain fresh cultures. Colonies of the test pathogens were scraped and suspended in 0.85% sterile NaCl solution. The test pathogen suspensions were adjusted to a turbidity equivalent to 0.1 OD at 625 nm before being spread onto MH agar plates. The suspension was adjusted to a turbidity equivalent to 0.1 OD at 625 nm, then spread evenly onto MH agar plates. Wells with a diameter of 9 mm were made in the agar using a sterile cork borer. The collected CFS from the bacterial culture was transferred into the wells, and the plates were incubated at 37 °C for 18 h. Cefoxitin (30 µg) and vancomycin (30 µg) (Sigma-Aldrich Co., St. Louis, MO, USA) were used as positive controls. The experiment was repeated in triplicate, and the mean ± SD of the inhibition zone diameters was recorded [58].

### 4.4. Production Kinetic Studies of Antimicrobial Compounds of FN33 Isolate

Only one isolate coded FN33 exhibited antibacterial activity against *S. aureus* and MRSA. A preculture of FN33 isolate was adjusted to 0.1 OD at 625 nm and inoculated at a 2% concentration in 50 mL of ZM broth. This culture was incubated at 30 °C with shaking at 150 rpm for 7 days. The CFSs were collected at 4, 8, 12, 16, 20, and 24 h on the first day, then once daily until the seventh day. Bacterial growth was monitored by measuring the OD at 625 nm, and the antibacterial activity of the CFS at each time point was evaluated using the agar well diffusion assay against *S. aureus* TISTR 517 and three MRSA strains. The experiment was conducted in triplicate and statistical analysis was performed using the Student’s *t*-test with a significance level of *p*-value < 0.05 to compare the antibacterial activity at different incubation times. The kinetics of antibacterial compound production were presented as mean ± SD [59].

### 4.5. Purification of the AMP and Amino Acid Sequence Determination

One-day-old cultures of FN33 colonies were suspended in 0.85% sterile NaCl solution. The bacterial suspension was adjusted to a turbidity equivalent to 0.1 OD at 625 nm. The bacterial suspension was used to prepare a 2% inoculum in 200 mL of ZM broth in a 1 L sterile Erlenmeyer flask. The 1 L total culture was incubated at 30 °C and 150 rpm for 48 h. The CFS was collected as described in the previous experiment. Ammonium sulfate was added stepwise to the collected CFS to achieve final saturation levels of 25%, 50%, and 75%. The precipitates from each saturation were collected by centrifugation at 18,000× *g* at 4 °C for 15 min. The collected precipitates were dissolved in deionized water before being desalted using a dialysis bag with a 3.5 kDa molecular weight cutoff membrane (SnakeSkin membrane, Pierce, Rockford, IL, USA). The dialysis was performed in deionized water at 4 °C for 16 h. Each dialysate was tested for antibacterial activity using the agar well diffusion assay against MRSA strain 2468. The dialysate showing antibacterial activity was subjected to cation-exchange chromatography using a HiTrap SP column (GE Healthcare Bio-Sciences AB, Uppsala, Sweden). A mobile phase consisting of 50 mM ammonium acetate (pH 5.0) and 50 mM NaCl was used. Isocratic elution was performed at a flow rate of 3 mL/min. The separated components were detected by UV absorbance at 214 nm and 280 nm. Fractions were collected based on the detected peaks, and each was evaluated for antibacterial activity prior to pooling the peak fractions. The pooled fractions were dialyzed using the same method described above before being further purified by reversed-phase chromatography (RPC). The fraction exhibiting antibacterial activity was subjected to RPC using an Inertsil ODS-3 C18 column (4.6 × 250 mm; GL Sciences, Tokyo, Japan) as a stationary phase. A gradient elution of mobile phase A (0.1% trifluoroacetic acid in deionized water) and mobile phase B (0.1% trifluoroacetic acid in 70% acetonitrile) at a flow rate of 1 mL/min was used for the separation of the antibacterial substance. The gradient elution was performed as follows: 0.0% mobile phase B for 25 min, 0.0% to 100% mobile phase B for 80 min. The peak signal was detected at 214 nm. Each fraction of 1 mL was collected from the chromatography and subsequently evaporated in a speed-drying vacuum concentrator (RVC 2-25 CDplus, Martin Christ, Osterode am Harz, Germany) to remove the residual acetonitrile and trifluoroacetic acid. The dried substance was reconstituted in deionized water to the same volume as the original collected fraction and subsequently tested for antibacterial activity using the agar well diffusion assay against MRSA strain 2468. The total volume of active fractions from each purification step was measured and dried by lyophilization (Gamma 2-16 LCSplus, Martin Christ, Osterode am Harz, Germany) before their weights were determined. The dried substances were reconstituted with sterile deionized water to the same volume as the volume before drying. The reconstituted fractions were prepared at varying concentrations through 2-fold dilution and assessed for antibacterial activity using the agar well diffusion assay against MRSA strain 2468. The arbitrary activity of each active fraction was calculated by raising the final dilution that produced an inhibition zone to the power of 2 and multiplying the result by 10. A purification balance table was constructed to evaluate purification efficiency based on the total volume, the total weight of the dried substance at each purification step, and the corresponding arbitrary activity. The purified peptides were analyzed by LC-MS/MS to investigate the peptide mass fingerprint (PMF) using an UltiMate 3000 liquid chromatography (LC) system coupled with high-resolution mass spectrometry (MS) (Thermo Fisher Scientific Inc., Waltham, MA, USA). Chromatographic separation was performed using a reversed-phase UHPLC column (4.6 × 30 mm; C18 Hypersil Gold, Thermo Fisher Scientific Inc., Waltham, MA, USA). A gradient elution method was employed, where the peptide sample was eluted from 0% to 100% of 0.1% formic acid in acetonitrile over 40 min at a flow rate of 300 µL/min. Mass spectrometry was conducted in both positive and negative ionization modes, using a spray voltage of 3.2 kV and a capillary temperature of 300 °C. The full mass spectra were acquired in the range of m/z 600–5000. Stepped normalized collision energy (NCE) was applied to optimize peptide fragmentation across a range of peptide lengths. De novo peptide sequencing was performed using the PEAKS Studio X software (Bioinformatics Solutions Inc., Waterloo, ON, Canada) [60]. Predicted physicochemical properties of the peptide sequences were obtained using the ProtParam tool via the Expasy website [61]. Sequence-based similarity searches were performed using antimicrobial peptide databases, including APD3 and DRAMP. Additionally, standard protein databases such as UniProtKB/Swiss-Prot and AlphaFold were used for structure-based similarity searches [62,63,64,65].

### 4.6. Determination of Minimum Inhibitory Concentration (MIC) and Minimum Bactericidal Concentration (MBC) of the AMP

The MIC and MBC of FN33 AMP were assessed against *S. aureus* TISTR 517 and three strains of MRSA. The broth microdilution method was performed following the guidelines of the Clinical and Laboratory Standards Institute (CLSI) [66]. Test strains including *S. aureus* TISTR 517 and MRSA strains 142, 1096, and 2468 were cultured on MH agar at 37 °C for 18 h. A single colony from each strain was suspended in 0.85% NaCl until it reached a turbidity of 0.1 OD at 625 nm (1 × 10^8^ CFU/mL). The bacterial cells were then diluted to 5 × 10^6^ CFU/mL using cation-adjusted Mueller–Hinton broth (CAMHB). Ten microliters of the cell suspension were added to each well of a 96-well plate to make the total volume in each well 100 µL. The AMP was added to achieve final concentrations ranging from 0.125 to 64 µg/mL. Vancomycin and cefoxitin were used as positive controls, while antibiotic-free samples were used as negative controls. The plate was incubated at 37 °C for 24 h. Each experiment was performed in triplicate. The MIC was determined as the lowest concentration that inhibited visible bacterial growth. For the MBC, 100 µL from each dilution was spread on MH agar and incubated at 37 °C for 24 h. The MBC was the lowest concentration at which no bacterial colonies appeared on the agar plates.

### 4.7. Effect of the AMP on Membrane Permeability

The Sytox Green uptake assay was performed to evaluate the disruption of cell membrane integrity induced by the AMP [67]. An overnight culture (16 h) of MRSA strain 2468 was harvested and washed three times with sterile PBS (pH 7.4) supplemented with 0.2% CAMHB as a diluent. The cell suspension was then diluted with the same diluent until the OD at 625 nm was 0.1 (1 × 10^8^ CFU/mL). An aliquot (100 µL) of the diluted cells was added to each well, followed by incubation with 10 µM Sytox Green (Thermo Fisher Scientific Inc., Waltham, MA, USA) in the dark for 15 min. The AMP was prepared at various concentrations, and 100 µL was added to each well to achieve final concentrations of 0.125×, 0.25×, 0.5×, 1×, and 2× MIC. Triton X-100 (AppliChem GmbH, Darmstadt, Germany) was used as a positive control and prepared at final concentrations ranging from 0.125% to 2%. These concentrations were used to compare the level of cell membrane permeability induced by the AMP. Membrane permeability was assessed by measuring fluorescence intensity using a microplate reader (Thermo Scientific Inc., Waltham, MA, USA) at excitation and emission wavelengths of 504 nm and 523 nm, respectively, over a 24 h period following AMP addition. Significant differences in fluorescence intensity (*p*-value < 0.05) were analyzed using two-way ANOVA and Tukey’s post hoc test for multiple comparisons between treated and untreated conditions.

### 4.8. Stability Analysis of FN33 AMP Under Various Environmental Conditions

The stability of FN33 AMP was analyzed under various environmental, enzymatic, and chemical conditions over incubation periods of 1, 6, and 12 h. The AMP solution was prepared in sterile purified water and adjusted to a final concentration of 32 µg/mL. To determine thermal stability, the AMP was exposed to temperatures of 30, 40, 60, 80, and 100 °C, as well as to autoclaving at 121 °C and 15 psi for 15 and 30 min. The susceptibility of the AMP to enzymatic degradation was examined by treating it with proteinase K, trypsin, and α-chymotrypsin (each at 1 mg/mL, Sigma-Aldrich, Warren, MI, USA), representing different proteolytic mechanisms. The AMP’s compatibility with surfactants was assessed by incubating it with 1% SDS, 1% CTAB, and 1% Triton X-100 (AppliChem GmbH, Darmstadt, Germany). Additionally, the effect of pH variations on AMP integrity was tested by adjusting the solution to pH 1, 4, 8, 10, and 14. The pH was neutralized to the original pH after incubation before conducting antibacterial activity tests. The antibacterial activity of FN33 AMP against MRSA strain 2468 was determined using the agar well diffusion assay, performed in triplicate. Stability data were expressed as residual activity percentages relative to the untreated sample (mean ± SD), and statistical significance was evaluated using two-way ANOVA and Tukey’s post hoc test, with a *p*-value < 0.05 considered significant.

### 4.9. Computational Modeling of the Secondary Structure of FN33 AMP

A computational approach was employed to model the structure and dynamics of FN33 AMP using MD simulations in an explicit solvent environment. The evolution of the peptide’s secondary structure was investigated based on its interactions with the solvent over the course of the simulation [68]. A single peptide molecule of FN33 AMP was built and saved in PDB format using BIOVIA Discovery Studio Visualizer version 21.1.0.20298 [69]. Structure preparation and parameterization with a CHARMM36 force field were performed using Visual Molecular Dynamics (VMD) version 1.9.3 [70,71]. The psfgen plugin version 2.0 was used to generate the peptide structure file [72], and the Solvate plugin version 1.5 was applied to introduce explicit water molecules into the simulation box [70]. MD simulations were carried out using NAMD version 3.0.1 under isobaric–isothermal (NPT) ensemble conditions, maintaining a constant number of atoms, pressure (1 atm), and temperature (310 K) using Langevin dynamics. Periodic boundary conditions were applied in all directions. The system underwent energy minimization prior to a 200 ns production run [73]. Peptide secondary structure transitions over the simulation time were analyzed using the Timeline plugin in VMD [74]. The dynamic 3D structure of the peptide in explicit water was generated from trajectory simulation data and visualized using VMD.

### 4.10. In Silico Analysis of FN33 AMP Dynamics in a Bacterial Membrane Model

The interaction between FN33 AMP and the bacterial membrane was supported by computational predictions. A molecular dynamics approach was employed to investigate the interaction between FN33 AMP and a simulated *S. aureus* cell membrane. The *S. aureus* membrane model was constructed using CHARMM-GUI Membrane Builder and consisted of 1,2-O-dipalmitoyl-sn-glycero-3-phospho-(1′-rac-glycerol) (DPPG) at 40%, 1,2-O-dipalmitoyl-sn-glycero-3-phospho-rac-(3-lysyl(1′-glycerol)) (Lysyl-DPPG) at 52%, and 1,1′,2,2′-tetramyristoyl cardiolipin (TMCL) at 8% [75,76]. The peptide and membrane systems were packed using Packmol version 21.0.1 [77]. The membrane was constructed as a planar bilayer, with the AMP initially positioned parallel to the lipid bilayer surface at a distance of 20 Å [78]. Explicit water molecules were used to solvate the system and Na⁺ counterions were added to neutralize the overall system charge. System preparation for MD production followed the same procedure used in the simulation of FN33 AMP’s secondary structure in bulk water. After running a 200 ns production simulation, the resulting trajectory file was analyzed using multiple tools. Visualization of AMP–membrane interactions was performed using VMD. The secondary structure of FN33 AMP during its interaction with the bacterial membrane was compared to its conformation in a bulk water environment to investigate membrane-induced structural changes [79]. MDAnalysis version 2.1, along with Python script version 3.9.0, was used to extract data on non-bonded interactions using a 4.5 Å cutoff, prior to plotting the interaction heat map [80,81,82]. The center-of-mass (COM) distance between the AMP and lipid components was analyzed over time to evaluate the binding dynamics and insertion depth. The membrane surface was defined by the polar headgroups of the phospholipids, while the lipid tails represented the hydrophobic hydrocarbon chains. The average depth of AMP insertion was determined relative to the entire phospholipid bilayer at the interaction site. To gain deeper insight into the interaction between FN33 AMP and the simulated bacterial membrane, the phospholipid molecules were analyzed by separating their head and tail regions. The COM of the lipid headgroups was used to define the membrane surface, which was then compared with the COM of FN33 AMP to assess surface binding. Additionally, the insertion depth of the AMP into the membrane was evaluated by measuring its COM relative to the COM of the lipid tails [78].

### 4.11. In Vitro Anti-Inflammatory Activity of FN33 AMP Assessed Using the Albumin Denaturation Assay

The anti-inflammatory effect of FN33 AMP was assessed using a modified albumin denaturation assay [83]. The reaction mixture consisted of 25 μL of egg albumin (derived from fresh hen eggs), 350 μL of PBS (pH 7.2, 0.1 M), and 250 μL of AMP or PBS (used as a reference). The mixtures were pre-incubated at 37 °C for 15 min, followed by denaturation at 70 °C for 5 min. After a 5 min cooling period on ice, the optical density (OD) at 660 nm was measured using a spectrophotometer. A five-point standard curve (500, 250, 100, 50, and 0 μg/mL) was prepared using diclofenac sodium (Merck Ltd., Bangkok, Thailand) as the reference drug and processed under the same conditions as the samples. The data were expressed as diclofenac sodium equivalents (μg/mL). The percentage inhibition of albumin denaturation was calculated using the following equation:

Inhibition of albumin denaturation (%) = [1 − (Absorbance_sample_/Absorbance_control_)] × 100

### 4.12. DPPH Free Radical Scavenging Activity of FN33 AMP

The antioxidant activity of FN33 AMP was assessed using the method described by Brand-Williams et al. (1995) [84]. A 200 μL sample or ascorbic acid (Gammaco Ltd., Bangkok, Thailand) was added to 2 mL of 0.1 mM DPPH solution (Merck Ltd., Bangkok, Thailand) and incubated at 25 °C in darkness for 30 min. The DPPH color change from purple to pale yellow was observed, and the absorbance at 517 nm was measured using a UV–visible spectrophotometer. The radical scavenging activity of FN33 AMP was determined from three independent experiments and expressed as IC_50_ values, calculated using the following formula:

Inhibition of DPPH (%) = [(Absorbance_control_ − Absorbance_sample_)/Absorbance_control_)] × 100

### 4.13. Bacterial Identification by Whole-Genome Sequencing

The FN33 isolate was cultured on ZM agar for 1 and 3 days to observe the colony morphology of vegetative cells and spores, respectively. Gram staining and malachite green endospore staining were performed to examine the morphology of vegetative cells and assess the spore-forming capability under a light microscope (Carl Zeiss, Oberkochen, Germany) [85,86]. Genomic DNA from FN33 was extracted and sequenced using the Illumina HiSeq platform in paired-end 150 bp mode (PE150; Illumina, San Diego, CA, USA), with sequencing services provided by U2Bio Co., Ltd. (Seoul, Republic of Korea). The genomic data were processed using the Galaxy Australia platform (version 23.1) [87]. Raw reads were quality-checked using FastQC (version 0.12.1) both before and after adapter trimming [88]. Low-quality reads were filtered and trimmed using Fastp (version 0.23.4), removing bases with lengths below 30 bp [89]. The FN33 genome was assembled de novo using SPAdes version 3.14.1, implemented via Shovill version 1.1.0 [90]. The quality and completeness of the assembled genome were evaluated using QUAST version 5.2.0 [91] and CheckM version 1.0.18 [92]. Species identification was verified using two complementary methods. Genome BLAST distance phylogeny (GBDP) was used to determine genomic similarity distances by comparing the FN33 genome sequence against the TYGS database using the BLAST algorithm [93]. Additionally, average nucleotide identity (ANI) was calculated to assess the sequence identity of fragmented genomic segments in comparison to reference genomes identified through the GBDP results [28]. A phylogenetic tree was constructed using reference genomes identified through the GBDP analysis to verify the closest taxonomic relationship to the FN33 genome. Fragmented genomic sequences of FN33 were compared with those of the closest taxonomic relative using FastANI version 1.1.0 and visualized using the Proksee web platform (https://proksee.ca, accessed on 20 January 2024). Genome annotation for identifying features and coding sequences (CDSs) was performed using Rapid Prokaryotic Genome Annotation (Prokka) version 1.14.6 [94]. Biosynthetic gene clusters (BGCs) associated with antimicrobial compounds were predicted using antiSMASH version 7.0 [95]. The predicted secondary metabolites from FN33 BGCs were compared to known reference BGCs available in the MIBiG version 3.1 and GenBank databases hosted by NCBI [96,97]. As part of the safety assessment of the FN33 isolate for microbial applications, antimicrobial resistance genes (ARGs) were identified. ARG prediction was conducted using the Resistance Gene Identifier (RGI) tool within the Comprehensive Antibiotic Resistance Database (CARD) [98]. Genomic features and related insights were further visualized using the CGViewBuilder tool (version 1.1.6) within the Proksee web service (https://proksee.ca, accessed on 20 January 2024). The circular genome map provided an in-depth view of annotated genes and genomic elements, with features organized into distinct color-coded tracks for clarity [99].

## 5. Conclusions

This study addresses the urgent need for new antimicrobial agents due to rising antibiotic resistance, particularly in MRSA. The marine-derived bacterium *Virgibacillus chiguensis* FN33 was found to produce a novel AMP with strong activity against *S. aureus* and MRSA by disrupting bacterial membranes. MD simulations supported the experimental findings by confirming the membrane-targeting mechanism of FN33 AMP. Genomic analysis identified biosynthetic gene clusters linked to AMP production. The peptide remained stable under physiological pH and temperature but was sensitive to proteolytic digestion, and its activity was influenced by surfactants, suggesting formulation considerations. In addition to its antimicrobial effects, FN33 AMP exhibited antioxidant and anti-inflammatory activities, indicating potential as a multifunctional therapeutic agent. Future studies should focus on toxicity, pharmacokinetics, and structural optimization to confirm its safety and efficacy. Overall, FN33 AMP shows strong promise for development in treating drug-resistant infections and for broader pharmaceutical and biomedical applications.

## Figures and Tables

**Table 1:** The CFS of FN33 culture exhibited antimicrobial activity against *S. aureus* TISTR 517 and MRSA strain 142, MRSA strain 1096, MRSA strain 2468, *E. coli* TISTR 887, *K. pneumoniae* TISTR 1383, and *P. aeruginosa* TISTR 357 using agar well diffusion method.

Tested Antimicrobial Agents	Zone of Inhibition (mm ± SD; n = 3)						
	*S. aureus* TISTR 517	MRSA Strain 142	MRSA Strain 1096	MRSA Strain 2468	*E. coli* TISTR 887	*K. pneumoniae* TISTR 1383	*P. aeruginosa* TISTR 357
CFS of FN33 culture	12.41 ± 1.20	17.47 ± 1.03	17.05 ± 1.83	17.98 ± 2.07	0.00 ± 0.00	0.00 ± 0.00	0.00 ± 0.00
Vancomycin (30 µg)	23.37 ± 1.27	24.13 ± 1.27	24.38 ± 0.51	24.64 ± 1.02	0.00 ± 0.00	0.00 ± 0.00	0.00 ± 0.00
Cefoxitin (30 µg)	35.87 ± 0.46	0.00 ± 0.00	0.00 ± 0.00	0.00 ± 0.00	35.12 ± 1.04	19.67 ± 0.88	0.00 ± 0.00

**Table 2:** The purification balance sheet of antibacterial substance from FN33.

Purification Procedures	Total Volume (mL)	Total Dried Weight (mg)	Arbitrary Activity (AU/mL)	Total Activity (AU)	Specific Activity (AU/mg)	Purification Factor (Folds)	Yield (%)
CFS of FN33 culture	976.86	1873.92	20	19,537.20	10.43	1.00	100.00
Protein precipitate of 50% ammonium sulfate saturation	45.24	86.25	80	3619.20	41.96	4.02	18.52
Active fraction of cation-exchange chromatography	17.38	11.06	80	1390.40	125.71	12.06	7.12
Active fraction of reversed-phase chromatography	3.22	0.84	320	1030.40	1226.67	117.66	5.27

**Table 3:** The MIC and MBC values of FN33 AMP. The standard antibiotics (vancomycin and cefoxitin) were used as the positive controls.

Tested Samples		*S. aureus* TISTR 517	MRSA Strain 142	MRSA Strain 1096	MRSA Strain 2468
FN33 AMP	MIC (μg/mL)	16	8	8	8
	MBC (μg/mL)	64	16	16	16
Vancomycin	MIC (μg/mL)	2	2	2	2
	MBC (μg/mL)	2	2	2	2
Cefoxitin	MIC (μg/mL)	2	>64	>64	>64
	MBC (μg/mL)	2	>64	>64	>64

**Table 4:** Antibacterial activity retention of FN33 AMP (%) against MRSA strain 2468 after incubation under various conditions.

Treatment Conditions	Antibacterial Activity Retention of FN33 AMP (%) Against MRSA Strain 2468 (Mean ± SD; n = 3)		
Control	1 h	6 h	12 h
	100.00 ± 0.84	100.00 ± 0.76	100.00 ± 0.42
Effect of temperature variation	1 h	6 h	12 h
30 °C	100.00 ± 0.66	100.00 ± 0.17	99.79 ± 0.47
40 °C	100.08 ± 0.48	100.02 ± 0.35	100.23 ± 0.28
60 °C	98.58 ± 1.74	93.53 ± 1.27 *^,†^	86.98 ± 0.79 *^,†^
80 °C	95.17 ± 0.43 *	90.91 ± 0.26 *^,†^	82.46 ± 0.55 *^,†^
100 °C	90.83 ± 0.67 *	83.38 ± 0.83 *^,†^	79.97 ± 0.06 *^,†^
Effect of autoclave condition	15 min		30 min
121 °C and 15 psi	72.82 ± 0.53 *		65.87 ± 0.82 *^,†^
Effect of protease digestion	1 h	6 h	12 h
FN33 AMP with proteinase K (1 mg/mL)	0.00 ± 0.00 *	0.00 ± 0.00 *	0.00 ± 0.00 *
FN33 AMP with trypsin (1 mg/mL)	99.60 ± 0.6	98.82 ± 0.67	99.04 ± 1.02
FN33 AMP with α-chymotrypsin (1 mg/mL)	0.00 ± 0.00 *	0.00 ± 0.00 *	0.00 ± 0.00 *
Effect of surfactant	1 h	6 h	12 h
FN33 AMP with CTAB (1% *w*/*v*)	93.19 ± 0.52 *	93.17 ± 0.48 *	92.51 ± 0.69 *^,†^
FN33 AMP with SDS (1% *w*/*v*)	117.08 ± 0.78 *	117.39 ± 0.96 *	115.95 ± 0.57 *
FN33 AMP with Triton X-100 (1% *w*/*v*)	81.23 ± 1.04 *	81.69 ± 0.38 *	81.30 ± 0.72 *
CTAB (1% *w*/*v*) alone	98.62 ± 0.69	98.89 ± 1.10	98.42 ± 0.72
SDS (1% *w*/*v*) alone	110.10 ± 0.84 *	109.30 ± 0.56 *	108.93 ± 0.57 *
Triton X-100 (1% *w*/*v*) alone	0.00 ± 0.00 *	0.00 ± 0.00 *	0.00 ± 0.00 *
Effect of pH variation	1 h	6 h	12 h
pH 1	74.56 ± 0.69 *	76.51 ± 0.39 *	72.85 ± 0.22 *^,†^
pH 4	97.42 ± 0.65 *	96.19 ± 0.21 *	95.30 ± 0.69 *^,†^
pH 8	99.53 ± 0.41	98.03 ± 0.65	98.76 ± 0.81
pH 10	74.63 ± 0.79 *	73.12 ± 1.04 *^,†^	69.46 ± 0.77 *^,†^
pH 14	68.58 ± 0.45 *	66.80 ± 0.27 *^,†^	65.80 ± 0.84 *^,†^

* Statistical significance of activity retention was determined by comparing treatments to the control within each time point using one-way ANOVA (*p*-value < 0.05). ^†^ Statistical significance of activity retention was determined by comparing different treatment times to the 1 h time point using one-way ANOVA (*p*-value < 0.05).

**Table 5:** Inhibition of albumin denaturation by FN33 AMP (mean ± SD; n = 3).

Group	Concentration (µg/mL)	% Inhibition of Albumin Denaturation
FN33 AMP	50	4.85 ± 0.93 *^,†^
	100	17.75 ± 0.97 *^,†^
	250	34.39 ± 1.82 *
	500	57.16 ± 1.06 ^†^
Diclofenac sodium (the concentration was equivalent to diclofenac)	50	3.09 ± 0.44 *^,†^
	100	12.24 ± 1.50 *^,†^
	250	25.31 ± 0.75 *^,†^
	500	34.85 ± 0.81 *^,†^

* *p*-value < 0.05 compared to FN33 AMP at a dose of 500 µg/mL, and ^†^ *p*-value < 0.05 compared to diclofenac sodium at a dose of 500 µg/mL.

**Table 6:** Antioxidant activity of FN33 AMP determined using the DPPH radical-scavenging assay (mean ± SD; n = 3).

Group	IC_50_ (µg/mL)
FN33 AMP	11.66 ± 2.87
Ascorbic acid	6.78 ± 1.58

## Data Availability

Data are contained within the article.

## Supplementary Materials

The following supporting information can be downloaded at: https://www.mdpi.com/article/10.3390/md23050209/s1, Figure S1: Mass Spectrometric Characterization and De Novo Sequencing of the Purified FN33 AMP.
