# Supplementary material for: Novel Anti-MRSA Peptide from Mangrove-Derived Virgibacillus chiguensis FN33 Supported by Genomics and Molecular Dynamics
Source: Mar Drugs. 2025 May 14;23(5):209. doi: 10.3390/md23050209 (PMC12113356; doi:10.3390/md23050209)
Supplement: Supplementary file 1 [file marinedrugs-23-00209-s001.zip › marinedrugs-3601543-supplementary.pdf]

Novel Anti-MRSA Peptide from Mangrove-Derived *Virgibacillus chiguensis* FN33 Supported by Genomics and Molecular Dynamics

Namfa Sermkaew [1,2], Apichart Atipairin [1,2], Phetcharat Boonruamkaew [1], Sucheewin Krobthong [3], Chanat Aonbangkhen [3,4], Jumpei Uchiyama [5], Yodying Yingchutrakul [6] and Nuttapon Songnaka [1,2,*]

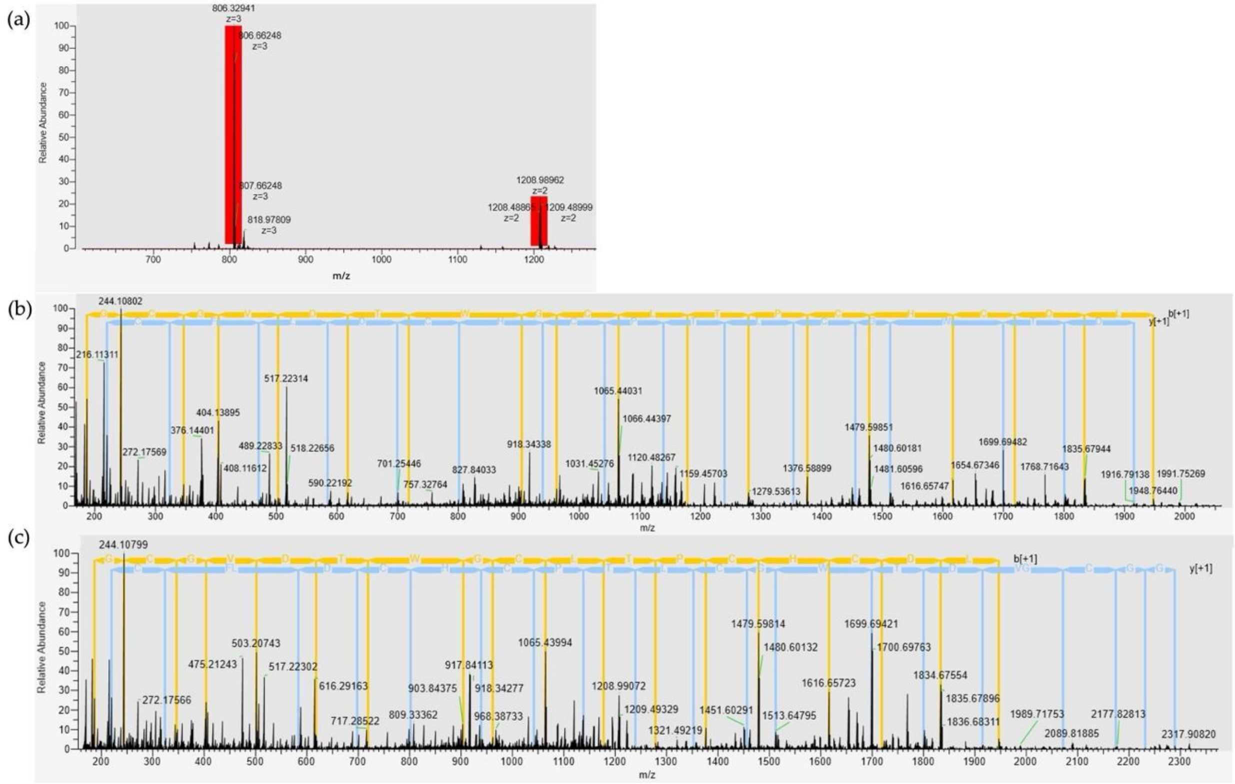

Figure S1. The full-scan mass spectrum of the purified AMP shows two major peaks at m/z 806.32941 Da ($[M+H]^{+3}$) and 1208.98962 Da ($[M+H]^{+2}$), confirming the molecular mass of FN33 AMP as 2416.98 Da. The amino acid sequencing by De novo analysis of secondary fragmentation of m/z 806.32941 Da ($[M+H]^{+3}$) (b) and 1208.98962 Da ($[M+H]^{+2}$) (c).
